# Occurrences of Organochlorine Pesticides along the Course of the Buffalo River in the Eastern Cape of South Africa and Its Health Implications

**DOI:** 10.3390/ijerph14111372

**Published:** 2017-11-10

**Authors:** Abdulrazaq Yahaya, Omobola O. Okoh, Anthony I. Okoh, Abiodun O. Adeniji

**Affiliations:** 1 SAMRC Microbial Water Quality Monitoring Center, University of Fort Hare, Alice 5700, South Africa; ookoh@ufh.ac.za (O.O.O.); aokoh@ufh.ac.za (A.I.O.); adenijigoke@gmail.com (A.O.A.); 2 Department of Chemistry, University of Fort Hare, Alice 5700, South Africa; 3 Applied and Environmental Microbiology Research Group, Department of Biochemistry and Microbiology, University of Fort Hare, Alice 5700, South Africa

**Keywords:** organochlorine, pesticides, pollutants, water, half-life

## Abstract

Most organochlorine pesticides (OCPs) which are increasingly used in agriculture and industry are not biodegradable and thereby persist in the environment for a very long period of time. They are capable of negatively impacting the health of humans and biota when present in a higher concentration than recommended. This study evaluated the concentrations of 17 OCPs in surface water samples collected from six sampling sites along the course of the Buffalo River in Eastern Cape, South Africa, between December 2015 and May 2016. The samples were subjected to solvent extraction, followed by florisil clean up, and analyzed using gas chromatography coupled with an electron capture detector. The individual concentrations of OCPs detected ranged from <LOD to 4403 ng/L in summer and <LOD to 313 ng/L in autumn. The levels of OCPs in the study area were generally above the United State Environmental Protection Agency (USEPA) limit of 100 ng/L in all the sampling locations in the two seasons. The cancer risk assessment values were below the permissible limit of the 10^−6^ level, although the life average daily dose were slightly above the USEPA maximum limits of 10^−4^. Therefore, there is a need for the adequate regulation of agrochemical storage, use, and disposal in this province and other parts of South Africa.

## 1. Introduction

Organochlorine pesticides (OCPs) have been extensively used in agriculture because of their insecticidal properties. They have been found to be potent for the control of ants, tsetse fly, termites, and even mosquitoes, amongst other insects. The products are now banned due to their high level of persistence in the environment [1]. For instance, p,p′-dichlorodiphenyltrichloroethane (DDT) is still detectable in nearly 10% of the population of the United States of America (USA) after 33 years of the prohibition, and its degradation product, dichlorodiphenylchloroethane (DDE), is found in almost every individual living in the region. Some of the products are still being distributed illegally in many countries with different trade names [2,3,4,5,6,7,8,9]. OCPs are ubiquitously found in almost every compartment of the environment owing to their chemical stability. They have a tendency to bioaccumulate in human and animal fatty tissues, as well as in some internal organs because of their hydrophobic and lipophilic properties [6,10,11]. Hence, they impact negatively on the health of humans, animals, and the environment at large [12].

Human exposure can be through inhalation, ingestion, or dermal contact, which might result in many toxicological health effects [2,3,4,5], including endocrine disruptions, reproduction and birth defects, immune system dysfunction, and cancer [13,14]. OCPs are efficiently transported into the aquatic systems by infiltration, runoff, and atmospheric deposition as a result of their volatilization [6,15,16]. They are found in the water systems both in dissolved and particle-bound forms. The freely dissolved forms of the organochlorine pesticides are easily bioavailable and are used for risk assessment in the aquatic environment [17]. Direct proportionality between the concentrations of the freely-dissolved pesticides in the aqueous phase and their fugacity and chemical activity, is a useful tool for the assessment of their fate in the environment [18]. They are generally detected in low concentrations in the water column (ng/L to pg/L). Hence, there is a need to adopt a very selective and sensitive method for their determination [6].

South Africa, a semi-arid region, is a front-line nation in the consumption of agrochemicals such as fertilizers and pesticides [5]. DDT is still being used for the eradication of malaria in no less than three of its provinces [19]. Notwithstanding, there is a dearth of data on the status of OCPs in the republic, even though South Africa is strongly industrialized and is also a signatory of the Stockholm Convention agreement on persistent organic pollutants (POPs) [6,20,21,22,23,24]. However, some studies were carried out on the occurrence, distribution, and fate of OCPs in the South African aquatic resources. For instance, Awofolu and Fatoki [21] determined the levels of OCPs in six selected freshwaters and sediments in Eastern Cape Province. The findings indicated that total OCP concentrations ranged from trace (2,4′-DDD) to 450 ng/L (β-BHC) in water samples and from trace (aldrin and 2,4′-DDD) to 184 × 10^3^ ng/kg (β-BHC) in sediments. The presence of some endocrine disrupting compounds like 2,4′-DDT, 4,4′-DDT, 2,4′-DDE, heptachlor, endosulfan, and the chlordanes was also reported.

Fatoki and Awofolu [21] also detected residues of OCPs in the marine, surface, ground, and drinking water samples collected from Sandile Dam, East London, and Port Elizabeth harbours, as well as Buffalo, Keiskammahoek, Tyume, and Swartskops Rivers in the Eastern Cape Province of South Africa. The analyses which were embarked upon with the intent to assess the effects of industrial and agricultural activities on the water bodies. The results revealed that pesticide residues in the aquatic systems were in the range of 5.5–160 ng/L, with 2,4′-DDD and HCB exhibiting the lowest and highest concentrations, respectively. A similar work on the subject includes the evaluation of concentrations of certain OCPs such as hexachlorocyclohexanes (HCHs), DDTs, DDDs, and DDEs in Hartbeespoort Dam across the four seasons in 2014 by Amdany et al. [6]. Results of the analyses showed that HCHs were detected in the range of 9.2 to 10.4 ng/L, while DDTs varied from 0.3 to 0.8 ng/L with the winter season recording the highest concentrations, probably due to the effects of atmospheric deposition. However, the lowest concentrations were observed in summer when the temperature was very high and the precipitation rate was low. 

Buffalo River traverses major towns and villages with a load of pollutants from domestic and industrial sources. Some impoundments are built on this river to supply water to nearby communities. One of the impoundments is the Laing Dam that supplies water to Zwelitsha, Bhisho, Berlin, and some parts of Mdantsane. The River receives toxic leachates containing heavy metals from a former tannery dump site near Zwelitsha. The East London harbor, situated in the Buffalo Estuary, is the final receiving point of the Buffalo River freshwater discharge. Severe pollution with elevated levels of heavy metals, bacteria (faecal and total coliforms), and contaminated run-off has resulted from human activities such as various operational spillages and ship repair, hence increasing the pollution status of the river that was formerly reported in the harbor area of the river. Final effluents containing several hazardous chemicals from some wastewater treatment plants and industries in the neighbourhood are reportedly discharged into the water body [25,26]. Previous studies conducted on the Buffalo River focused on the physico-chemical qualities of its water [27,28] and the status of POPs in the river water over a decade ago [20]. Recently, the total petroleum hydrocarbon profiles of both the water and sediment of the Buffalo estuary were determined by Adeniji et al. [29]. However, there is a paucity of data on the current levels of OCPs in the Buffalo River. This study therefore aimed at determining concentrations of 17 OCPs from six sampling sites along the course of the Buffalo River in the Eastern Cape province of South Africa for two seasons with a view to assess the current health status of the water body and risk of exposure to these contaminants in the aquatic system.

## 2. Materials and Methods

### 2.1. Description of Study Sites

**Figure 1:**
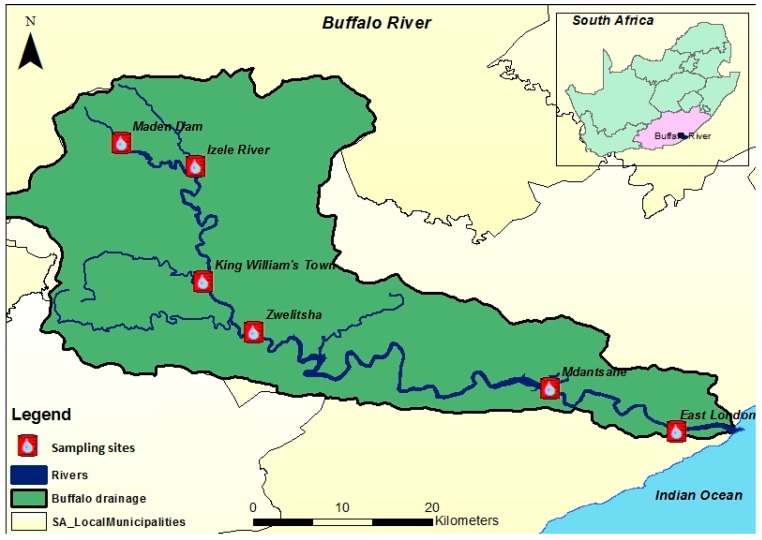
Map of Buffalo River showing the sampling sites for this study.

Buffalo River’s source can be found at the Amathole hill and flows via Maden dam, Izele, King William’s town, Zwelitsha, and Madnsatne, and finally empties into the Buffalo estuary in East London City, Eastern Cape Province (Figure 1). Along its course, there are a number of dams including Laing, Bridle Drift, Rooikrantz, and Maden dams providing water for domestic and agricultural purposes.

### 2.2. Field Sampling

The co-ordinates of the sampling sites are shown in the Table 1. One litre amber glass bottles used for sample collection and other glass vials were washed with soap and rinsed with a sufficient quantity of tap water. They were dried in an oven at 105 °C overnight, cooled, rinsed with acetone, drained, and dried again in the oven at 105 °C for another two hours. The glass vials used were all covered with PTFE lined lids.

Grab samples of surface water were collected in triplicate from a 100 mm depth below the water surface and placed in the pre-cleaned amber bottles from each of the six sampling sites. Samples were collected in the morning (7 a.m.–10 a.m.) from December 2015 to May 2016 and preserved with 5 mL of hydrochloric acid (1:1). They were stored in ice-chests at 4 °C and transported immediately to the laboratory for analysis [6,29,30,31].

Water completely dried off IZ and partly some sampling points of KWT and ZW, so sampling could not be carried out in winter.

### 2.3. Chemicals

The Organochlorine pesticides (OCPs) standard mixture (100 μg/mL in 1 mL) was made up of alpha Benzenehexachloride (α-BHC), gamma-Benzenehexachloride (γ-BHC), beta-Benzenehexachloride (β-BHC); Heptachlor; Delta-Benzenehexachloride (δ-BHC); Aldrin; Heptachlor Epoxide, Endosulfan I, 4,4-DDE, Dieldrin; Endrin, 4,4-DDD; Endosulfan II, 4,4-DDT, Endrin Aldehyde, Endosulfan Sulphate, and Methoxychlor. Decachlorobiphenyl (DCBP) of 1000 μg/mL in 1 mL (stock solution) was used as the surrogate standard in accordance with EPA Method 8081B [31]. 

All the standards were purchased from Ultra Scientific Analytical Solution, (North Kingstown, RI, USA), while the HPLC grade solvents (n-Hexane, dichloromethane and acetone) were obtained from Sigma Aldrich (Praha, Czech Republic). Merck (Darmstadt, Germany) supplied the sulphuric acid (99% purity) for this project. Ultra-pure nitrogen and helium gases (99.99%) were purchased from Afrox Limited (Gauteng, South Africa), and were used as the make-up and carrier gases for the gas chromatography (GC), respectively. The stock standards were diluted to 20 μg/mL with acetone: toluene (1:1) and were kept in the refrigerator at <4 °C as working standards for subsequent use. Calibration standards with inclusion of the surrogate standard were prepared by serial dilution with the same solvent mixture in the range of 10 to 600 ng/L for instrument calibration. Silica gel used for the column cleanup was activated in the oven at 130 °C for 24 h and cooled in a desiccator before use [32].

### 2.4. Preparation of Sample

Each of the surface water samples (500 mL) collected from the study sites was spiked with 1 mL of 10 μg/mL surrogate standard and extracted three times with 30 mL portions of dichloromethane in a 1 L separating funnel in all locations [33,34], except those obtained from Buffalo Estuary, which were poorly extracted with dichloromethane. The poor extraction could be linked with matrix effects brought about by a higher salinity level in the water body. The extraction of water samples from the estuary was better with a mixture of hexane and acetone (1:1 *v*/*v*) and was therefore used for subsequent extraction of water from the site [35] All sample extracts were combined, dried with anhydrous sodium sulphate and concentrated to about 3 mL in a rotary evaporator, solvent exchanged with 40 mL of n-hexane, re-concentrated to approximately 1 mL, and subjected to column clean up as detailed below [31,36,37]. 

### 2.5. Clean-Up

Each extract was passed through a glass column (10 mm I.D. × 30 cm) packed with 5 g florisil, with a 2 g layer of anhydrous sodium sulphate on top. The column was pre-eluted with 10 mL n-hexane. OCPs were subsequently eluted with 40 mL n-hexane and concentrated at 37 °C using a rotary evaporator to approximately 2 mL [31,38,39].

### 2.6. Instrumental Analysis

The analyses of OCPs were carried out with an Agilent 7820A GC (Agilent Technologies, Johannesburg, South Africa) coupled with micro-ECD (Model G2397AE, Santa Clara, CA, USA). Helium was used as the carrier gas at a constant flow rate of 3.5 mL/min. One microliter (µL) of extract was injected in splitless mode at 250 °C into an HP-5 capillary column (30 m × 0.25 mm × 0.25 μm). The oven was programmed to start at 50 °C, then ramped at 40 °C/min to 150 °C, and finally at 5 °C/min to 250 °C. The runtime was 22.5 min, while the ECD temperature was set at 300 °C. Nitrogen was used as the make up gas at 30 mL/min [40]. 

The instrument was calibrated with the prepared working standards for the 17 OCPs with concentrations ranging from 10 to 600 ng/L. The graphs were linear, having correlation coefficients varying from 0.9884 to 0.9975 (Table 2), which were within the acceptable range of *r*^2^ ≥ 0.990 [41] A response factor was generated for each analyte compound from the calibration curves by means of Agilent Chemstation software [28]. Analytes in the sample extracts were identified by comparing their retention times with those of their standards and quantification was done using the instrument’s software [42,43,44]. 

### 2.7. Quality Assurance

The quality assurance measures taken included the blanks analysis of Milli-Q water in the same manner as the samples, as well as triplicate analyses of the field samples [33,40] All samples were spiked with the surrogate (DCBP) as to check the extraction efficiency and the percentage recovery was calculated. Milli-Q water was spiked with 60 ng/L of OCPs standard and the percentage recovery of each analyte was calculated in accordance with the method of Kumar et al. [45]. 

Limit of detection (LOD) was determined by running a standard mixture, containing 100 ng/L of each analyte compound, through the instrument seven times. The signal to noise ratio (SN) was determined by division of the mean value with standard deviation of the results obtained for the replicate analyses and was found to be greater than 5, as suggested by WDNR [46]. However, the LOD was estimated by multiplying the “*t*” value at a 99% confidence level with standard deviation (δ) of the instrument response. Similarly, the limit of quantitation (LOQ) was computed as 10δ and relative standard deviation (RSD) as the percentage ratio of δ to the mean value [46,47].

### 2.8. Statistical Analysis

The one way analysis of variance (ANOVA), regression analysis, mean, and standard deviation of the data generated were determined using MINITAB for windows version 12.11 (2014, Minitab Ltd., University City, PA, USA) at a significance of *p* < 0.05.

### 2.9. Risk Assessments

The risk level of the analytes was assessed using the life average daily dose (LADD), cancer risk, and hazard quotient (HQ) [36,48,49,50]. Values were evaluated from Equations (1)–(4) in accordance with standard methods [51,52,53,54]. 

For calculation of the risk assessment:

(1) $$HQ=\frac{ADD}{RfD}$$

where: HQ = Hazard Quotient (no unit); ADD = intake exposure level (mg/kg/day); RfD = Reference Dose (mg/kg/day).

(2) $$ADD=\frac{C\times FI\times IR\times EF\times ED}{BW\times AT}(\mathrm{mg}/\mathrm{kg}/d)$$

(3) $$LADD=\frac{C\times FI\times IR\times EF\times ED}{BW\times AT}(\mathrm{mg}/\mathrm{kg}/d)$$

ADD = intake exposure level mg/kg/day; LADD = Life average daily dose (mg/kg/body weight) C = Average concentration of the analyte (OCPs) during the monitoring periods (mg/L); FI = Fraction ingested (an absolute number with 0–1, but previous studies estimated FI = 0.98); IR = Daily water intake based on age group; Age 0–6 years = 0.3 L/day, Age 7–17 years = 1 L/day; Adult = 1.4 L/day; EF = Exposure Frequency = 365 days/year; ED = Exposure duration based on age group; Age 0–6 years = 6; Age 7–17 years = 11; Adult = 3; BW = Average body weight. Age 0–6 years = 30 kg; Age 7–17 years = 46 kg; Adult = 70 kg. AT = Averaging times in days; AT_0–6_ = 2190 days; AT_7–17_ = 4015 days; AT_Adult_ = 10,950 days. 

Note: For LADD, the AT = 70 years × 365 = 25,550 days (the same for all age groups).

(4) $$Cancer\;risk=\frac{C\times DI\times ED\times CSF\times CF}{BW\times AT}$$

C = concentration of OCPs during the monitoring period (mg/L), DI = daily input L day^−1^: 2 L day^−1^; ED = Exposure duration (Year): 30 years, BW = body weight (kg): 60 kg; AT = Average life span (year): 70 years × 365 = 25,550 days (for all age group); CSF = Cancer slope factor (mg/kg/day): 0.07 (mg/kg/day); CF = Conversion factor: 10^−6^.

Note: The average time (AT) of this research was 183 days.

HQ > 1.0 indicates that the compounds pose a potential threat to ecosystems or that harmful effect may arise; HQ < 1.0 shows a relatively low risk. A larger HQ implies a greater ecological risk. ADD > 10^−4^ shows the maximum lifetime cancer risk while LADD > 10^−6^ suggests the highest risk of cancer, but LADD = 10^−3^ indicates that protective measures are required. Similarly, a cancer risk value > 10^−6^ suggests there is the highest cancer risk; however, values equal to 10^−3^ require protective measures [55,56].

## 3. Results and Discussion

### 3.1. Results

#### 3.1.1. Quality Assurance

**Figure 2:**
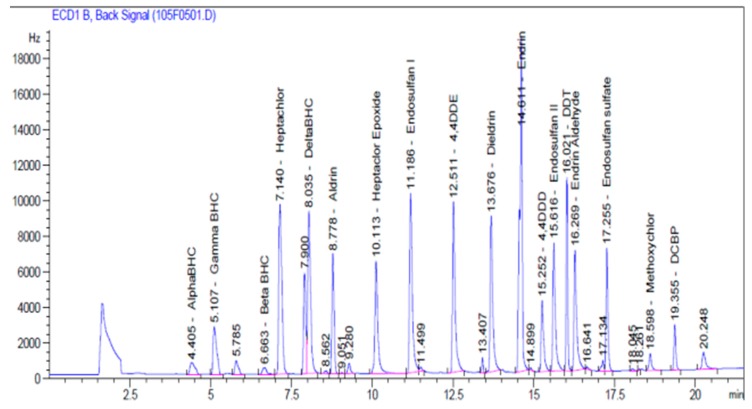
Typical chromatogram of the OCP standard.

The recoveries of OCPs from Milli-Q water ranged from 80 to 117%, while the surrogate recoveries from the samples were largely between 76% and 93%, except in a few cases at the estuary where they were found to be a little lower (≥57%), which could possibly be a result of the sample matrix. The LOD, LOQ, and RSD for OCPs varied from 20 to 60 ng/L, 110 to 530 ng/L, and 2 to 6%, respectively. The retention times, correlation coefficients (*r*^2^), and linear equations for the standard mixtures are shown in the Table 2. A typical chromatogram of the OCP standard mixture is shown in Figure 2.

#### 3.1.2. Level of OCPs in the Buffalo River

The number of OCPs detected at each of the six sampling points viz BRE, MSN, ZW, KWT, IZ, and MD in summer were 11, 11, 14, 8, 5, and 3, respectively (Table 3). The concentrations of OCPs varied from < LOD to 4403 ng/L, with the maximum concentration recorded at KWT. The most frequently detected in descending order among the OCP congeners in summer are aldrin, heptachlor epoxide > endosulfan I > 4,4-DDD, β-BHC, methoxychlor > α-BHC, heptachlor, δ-BHC > dieldrin, endosulfan II, endrin aldehyde > γ-BHC, 4,4-DDE, edrin, 4,4-DDT.

However, in autumn, the occurrence of α and β-BHC were 100% in all the sampling locations except at MD (Table 4). The individual concentrations in the season varied between <LOD and 313 ± 0.06 ng/L, with the highest obtained from MSN, whereas the total concentrations were in the range of 183–978 ng/L. The frequency of detection of the most predominant among the organic contaminants in autumn was in the order: α-BHC, β-BHC > aldrin, methoxychlor > heptachlor epoxide, 4,4-DDD > γ-BHC, heptachlor, endosulfan I, endosulfan II, 4,4-DDT > δ-BHC 4,4-DDE, endrin aldehyde.

The cancer risk assessment results, ADD, LADD, and hazard quotients are shown in Table 5 and Table 6. The cancer risk values, ADD, and hazard quotients were found to be lower than the maximum allowable limits whereas LADD was above the permissible levels for OCPs in polluted water as recommended by USEPA [48,51,53,56,57].

### 3.2. Discussion

#### 3.2.1. Quality Assurance

The limits of quantitation (LOQ) and detection (LOD) were less than 0.1 μg/mL and relative standard deviation (RSD) was below 20% for all the analytes and compares favourably with similar studies carried out elsewhere [45,58,59,60].

#### 3.2.2. Level of OCPs

The target compounds were selected based on their intensive use in agriculture in the Eastern Cape Province, South Africa. The concentrations of OCPs detected in all the study sites were higher in summer than autumn. This is expected because of the heavy rainfall that the country experiences during the season. Runoff from the agricultural farmlands along the flow course of the river could be a principal contributor to the high concentrations of OCPs in the study areas. Another possible reason for the high concentration in summer is the volatilization of the chemicals when applied and the subsequent dry and wet deposition into the water body [5,6]. The concentrations recorded at the Buffalo estuary could also be linked with the influx of contaminants from its influent rivers/creeks and drainage channels from the East London city, as well as industrial and municipal waste discharge into the aquatic environment. There is also the possibility of leaching of solid wastes from a closed landfill site near Creek 2 into the estuary water, which might be increasing the levels of these organic pollutants in the water system [25].

In summer, the non-detectable target compounds were γ-BHC, heptachlor, 4,4-DDE, and endrin aldehyde, which could possibly have been converted to α-BHC, heptachlor epoxide, DDD or methoxychlor, and endrin, respectively, under favorable environmental conditions such as lower pH values, temperatures, and redox reactions [61,62]. The high concentration of methoxychlor, an important raw material for the production of certain insecticides and herbicides, could be associated with its low conversion through photo-oxidation [61,63,64]. γ-BHC, dieldrin, and endosufan II were detected at very low concentrations in summer, suggesting possible degradation to α-BHC, Eldrin, and/or endosulfan suplhate, respectively, at MSN.

Buser and Muller [65] reported that pesticides such as γ-HCH in an active sewage sludge could be converted into α- or δ-HCH into a certain percentage at a slow rate under anaerobic circumstances. Hence, the high concentrations of OCPs recorded in summer at ZW could be attributed to the indiscriminate discharge of wastes from sludge, aerated treatment ponds, industries, households, the tannery, and textile mill [66,67]. The organic pollutants were less prone to volatilization in autumn than summer, probably because of their high molecular weights and lower temperature of the river water in the season, hence the levels in autumn were low compared to the previous season [66,67]. All the analytes were found to be present at KWT in autumn while fewer were detected in summer. Detection of all the analytes in KWT was an indication that some agricultural farms were located in that area and that a substantial dose of pesticides applied on the crops subsequently drained into the river when it rained [4,62,68]. The concentrations recorded at this sampling location were one hundred times higher than those reported by Fatoki and Awofolu [40]. 

In autumn, more of the target compounds, α and β-BHC, aldrin, heptachlor expoxide, DDT, and methoxychlor were detected at IZ than in summer, probably due to the influx from the Amadelakufa co-operative farm and animal husbandry, as well as domestic wastes from Izele town, which drained into the river [69,70,71]. At MD during the summer, few target compounds were detected whereas all were detected but at very low concentrations in autumn. Though, the Maden dam was said to be pristine, therefore presenting the lowest concentrations of pesticides in both seasons. The little that was obtained at this study site could be due to long range transportation of the pesticides by air currents from agricultural farmlands in the neighbourhood and other anthropogenic activities at the nearby Izele town and its suburbs [72,73,74].

OCPs such as DDT, DDE, heptachlor, endosulfans, and chlordanes are regarded as endocrine disrupting chemicals [5]. DDT is known to decompose into DDE and DDD, which also possess similar toxicological properties as the parent compound. The presence of these chemicals in the river water could impact negatively on humans and animals in whose fatty tissues and internal organs they accumulate. The higher values of 4,4-DDD and 4,4-DDE than the 4,4-DDT suggests possible degradation of the parent compounds into metabolites, thereby increasing their levels in the water milieu [9,15]. The rate at which heptachlor epoxide, endosulphan sulphate, and HCHs degrade is very slow. This explains why some of them were detected in both seasons because they have a tendency to stay in the environment longer than many others [75].

The concentrations recorded in this study were comparable to similar findings reported for water samples from Aposelemis, Greece [63]; Afyonkarahisar, Turkey [76]; and the Arctic region of China [65]. However, they were found to be much lower compared to other research works carried out in Agboyi Creek, Lagos, Nigeria [77] and Jukskei River, Gauteng, South Africa [5]. Notwithstanding, the concentrations of OCPs in the two seasons of study were found to be higher than the USEPA maximum permissible limit of 100 ng/L [78]. Hence, there is a need for strict compliance which should be facilitated by the enforcement of relevant environmental laws in the area.

#### 3.2.3. Risk Assessment

Risk is the possibility that a receptor could develop cancer in their life time based on the exposure and the toxicity of pollutants. Risk evaluation encompasses the calculation of the upper limit in excess of life cancer risk and non-carcinogenic hazard of a receptor [79]. The health risks assessment in this study was based on the assumption that living organisms might be exposed to these pollutants in the river water by ingestion, dermal contact, or inhalation. Factors that could increase one’s risk of having cancer are age, habits, health conditions, inherited cancer syndrome, and environments such as exposure to harmful chemicals and hazardous substances. Hence, the following carcinogenic and non-carcinogenic indices were used for the assessment of health risk in this study: HQ, ADD, LADD, and cancer risk values [15,48,49,51,53,54,55,57].

Hazard Quotient is the ratio of the predicted environmental concentration (health exposure) to predicted-no-effect concentration (no health effect) [49]. Reference dose (RfD) is the daily oral intake of water based on the weight of human body that is lower than the level considered to be no-health effects or zero health risk on humans over a life time, measured in mg/kg/day by USEPA. This assumes that a body of 70 kg takes two liters of water on daily basis [55]. Average daily dose (ADD) is the level of intake of the contaminant per day (mg/kg/day), while life average daily dose (LADD) is the intake of pollutant that is contained in drinking water averaged over the lifetime and measured in mg/kg/body weight [15,49].

With reference to Table 5 and Table 6, the HQ and ADD values calculated for the OCPs in the river water were lower than the acceptable risk level, thereby suggesting that the contaminants are not likely to cause harmful non-carcinogenic health and ecological effects in the population. Also, since the cancer risk values were generally below the permissible limit, there is no likelihood of any cancer risk for the consumers of the surface water [15,55,56,80,81]. However, the LADD was slightly higher than the expected value of 10^−4^, indicating that there is a slight possibility of lifetime health risk [80,82]. 

## 4. Conclusions

The surface water of the Buffalo River in Eastern Cape, South Africa was evaluated for OCPs along its flowing course using liquid-liquid extraction and GC-ECD for the instrumental identification and quantification. Generally, total concentrations of the contaminants were higher in summer than autumn possibly due to many factors including runoff, industrial and municipal waste discharge, volatilization and deposition of the chemicals, and influx from tributaries, amongst many others. Several OCPs including the endocrine disrupting chemicals like DDE, DDD, and DDT were determined in this study. Similarly, the high concentrations of compounds such as aldrin and heptachlor epoxide in some locations could be due to their percentage in the formulation of the pesticides used in the East Cape Province of South Africa. Hence, the levels of these pollutants detected in the river water constituted a threat to the aquatic, wild, and human lives and should be controlled. Therefore, the set guidelines for the use and disposal of agrochemicals in the country should be enforced and a regular assessment of the water quality parameters should be sustained.

## Figures and Tables

**Table 1:** Co-ordinates of the sampling points.

Sampling Site	Latitude	Longitude	Description of Sites
Buffalo River Estuary (BRE)	33°1′26.06″ S	27°53′26.41″ E	Municipal and industrial effluent discharged, including agricultural run-offs.
Mdantsane (MSN)	32°58′50.94″ S	27°42′28.78″ E	Sewerage works, heaps of refuse at the dump and the Potsdam treatment works and food factories.
Zwelitsha (ZW)	32°55′48.97″ S	27°25′59.31″ E	Influx of wastes from agricultural farm lands, refuse dumpsites, sewerage outfalls and aerated treatment pond.
King William’s Town (KWT)	32°52′44.06″ S	27°22′54.89″ E	Hazardous industrial and domestic wastes as well as agricultural run-offs are discharged.
Izele River (IZ)	32°45′52.32″ S	27°22′22.42″ E	Domestic wastes and agricultural run-offs are discharged.
Maden Dam (MD)	32°44′26.15″ S	27°17′59.47″ E	Pristine but at times cattle grazing

**Table 2:** Retention time, equation, and correlation coefficient (*r*^2^) of OCPs.

OCPs	Retention Time (min)	Equation	*r*^2^
α-BHC	4.405	*y* = 13833*x*	0.9951
γ-BHC	5.107	*y* = 64859*x*	0.9956
β-BHC	6.663	*y* = 99379*x*	0.9928
Heptachlor	7.140	*y* = 207701*x*	0.9886
δ-BHC	8.035	*y* = 186188*x*	0.9924
Aldrin	8.778	*y* = 107562*x*	0.9938
Heptachlor epoxide	10.113	*y* = 130345*x*	0.9887
Endosulfan I	11.186	*y* = 192696*x*	0.9962
4,4-DDE	12.511	*y* = 172980*x*	0.9937
Dieldrin	13.676	*y* = 155561*x*	0.9937
Endrin	14.611	*y* = 326806*x*	0.9969
4,4-DDD	15.252	*y* = 64298*x*	0.9949
Endosulfan II	15.616	*y* = 124856*x*	0.9884
4,4-DDT	16.021	*y* = 118677*x*	0.9975
Enrin Aldehyde	16.269	*y* = 129680*x*	0.9916
Endosulfan Sulfate	17.255	*y* = 95893*x*	0.9913
Methoxychlor	18.598	*y* = 18172*x*	0.9959
DCBP	19.355	*y* = 35257*x*	0.9886

**Table 3:** Total mean concentrations (ng/L) of OCPs in summer in surface water along the course of the Buffalo River (Values are means ± SD; N = 3).

OCPs	Sampling Points												Range
	BRE	FD	MSN	FD	ZW	FD	KWT	FD	IZ	FD	MD	FD	
α-BHC	446 ± 0.11	100	684 ± 0.27	100	1476 0.08	33	<LOD	0	<LOD	0	<LOD	0	<LOD–1476
γ-BHC	<LOD	0	<LOD	0	127 ± 0.01	33	<LOD	0	<LOD	0	<LOD	0	<LOD–127
β-BHC	170 ± 0.04	100	654 ± 0.56	67	218 ± 0.07	100	4403 ± 0.02	33	<LOD	0	<LOD	0	<LOD–4403
Heptachlor	<LOD	0	79 ± 0.01	100	31 ± 0.03	33	40 ± 0.01	33	<LOD	0	<LOD	0	<LOD–79
δ-BHC	34 ± 0.01	33	56 ± 0.01	100	78 ± 0.03	67	46 ± 0.01	100	<LOD	0	<LOD	0	<LOD–78
Aldrin	243 ± 0.03	100	100 ± 0.04	100	253 ± 0.10	100	117 ± 0.06	100	197 ± 0.08	67	120 ± 0.04	100	100–253
Hep. Epoxide	292 ± 0.11	100	54 ± 0.02	100	56 ± 0.01	100	50 ± 0.01	100	194 ± 0.12	100	151 ± 0.16	100	50–292
Endosulfan I	48 ± 0.01	100	43 ± 0.02	100	63 ± 0.02	67	57 ± 0.01	33	389 ± 0.01	33	<LOD	0	<LOD–389
4,4-DDE	<LOD	0	<LOD	0	234 ± 0.01	67	<LOD	0	<LOD	0	<LOD	0	<LOD–234
Dieldrin	86 ± 0.02	100	<LOD	0	<LOD	0	<LOD	33	<LOD	0	<LOD	0	<LOD–86
Endrin	<LOD	0	<LOD	100	<LOD	0	<LOD	0	<LOD	0	<LOD	0	<LOD
4,4-DDD	34 ± 0.01	100	494 ± 0.01	67	121 ± 0.12	67	<LOD	0	311	33	<LOD	0	<LOD–494
Endosulfan II	30 ± 0.01	100	<LOD	0	60 ± 0.03	67	<LOD	0	<LOD	0	<LOD	0	<LOD–60
4,4-DDT	<LOD	33	<LOD	67	218 ± 0.12	67	<LOD	33	<LOD	33	<LOD	0	<LOD–218
Endrin Alde.	<LOD	0	208 ± 0.01	33	<LOD	0	<LOD	33	<LOD	0	<LOD	0	<LOD–208
End. Sulphate	174 ± 0.03	100	440 ± 0.06	67	571 ± 0.07	100	381 ± 0.12	67	392 ± 0.04	100	<LOD	100	<LOD–571
Methoxychlor	2080 ± 0.04	100	113 ± 0.01	33	576 ± 0.09	100	<LOD	0	<LOD	0	164 ± 0.01	33	<LOD–2080
∑OCPs	3637 ± 0.42	-	2525 ± 0.99	-	4148 ± 1.90	-	5094 ± 0.26	-	1483 ± 0.26	-	435 ± 0.21	-	435–5094
No. of OCPs	11	-	11	-	14	-	8	-	5	-	3	-	

LOD: Limit of detection. Hept.: Heptachlor, Alde.: Aldehyde, End.: Endosulfan. FD: Frequency of detection (%).

**Table 4:** Total concentration (ng/L) of OCPs in autumn in surface water along the course of Buffalo the River (Values are means ± SD; N = 3).

OCPs	Sampling Points												Range
	BRE	FD	MSN	FD	ZW	FD	KWT	FD	IZ	FD	MD	FD	
α-BHC	125 ± 0.04	100	313 ± 0.06	100	224 ± 0.3	100	293 ± 0.26	100	187 ± 0.24	100	39 ± 0.02	33	39–313
γ-BHC	<LOD	0	<LOD	0	63 ± 0.01	33	<LOD		<LOD		<LOD	0	<LOD–63
β-BHC	130 ± 0.07	100	119 ± 0.01	100	62 ± 0.4	100	53± 0.04	100	127 ± 0.03	100	87 ± 0.01	33	53–130
Heptachlor	127 ± 0.01	33	<LOD	0	<LOD	0	<LOD	0	93 ± 0.08	67	<LOD	0	<LOD–127
δ-BHC	<LOD	33	<LOD	0	<LOD	0	<LOD	0	<LOD	0	<LOD	0	<LOD
Aldrin	30 ± 0.02	100	85 ± 0.07	67	<LOD	0	97 ± 0.08	67	143 ± 0.11	67	57 ± 0.01	67	<LOD–143
Hep. Epoxide	<LOD	0	<LOD	0	86 ± 0.05	33	29 ± 0.01	100	21 ± 0.01	67	<LOD	0	<LOD–86
Endosulfan I	<LOD	0	31 ± 0.01	67	<LOD	0	<LOD	0	60 ± 0.02	100	<LOD	0	<LOD–60
4,4-DDE	<LOD	0	<LOD	0	201 ± 0.01	67	<LOD	0	<LOD	0	<LOD	0	<LOD–201
Dieldrin	<LOD	0	<LOD	67	<LOD	0	<LOD	0	<LOD	0	<LOD	0	<LOD
Endrin	<LOD	0	<LOD	0	<LOD	0	<LOD	0	<LOD	0	<LOD	0	<LOD
4,4-DDD	36 ± 0.02	67	79 ± 0.06	67	<LOD	0	<LOD	0	53 ± 0.03	67	<LOD	0	<LOD–79
Endosulfan II	<LOD	0	222 ± 0.02	67	154 ± 0.02	33	<LOD	0	<LOD	67	<LOD	0	<LOD–222
4,4-DDT	<LOD	0	44 ± 0.03	67	<LOD	0	<LOD	0	40 ± 0.26	67	<LOD	0	<LOD–44
Endrin Alde.	<LOD	0	<LOD	0	<LOD	0	<LOD	0	<LOD	0	<LOD	0	<LOD
End. Sulphate	<LOD	0	48 ± 0.04	100	<LOD	0	<LOD	0	<LOD	67	<LOD	0	<LOD–48
Methoxychlor	236 ± 0.4	33	39 ± 0.3	100	89 ± 0.07	67	185 ± 0.16	0	252 ± 0.2	0	<LOD	0	<LOD–252
∑OCPs	684 ± 0.54	-	978 ± 0.69	-	878 ± 0.87	-	657 ± 0.55	-	976 ± 0.75	-	183 ± 0.04	-	183–978
No. of OCPs	6	-	9	-	7	-	6	-	9	-	3	-	

LOD: Limit of detection. Hept.: Heptachlor, Alde.: Aldehyde, Endo.: Endosulfan. FD: Frequency of detection.

**Table 5:** Hazard Quotient (HQ) of OCPs for age 0–6, 7–17 years, and adult.

OCPs	HQ_0–6_ × 10^−6^	H_7–17_ × 10^−6^	HQ_Adt_ × 10^−6^
γ-BHC	7	5	1
Heptachlor	55	33	11
Aldrin	2013	1193	403
Heptachlor Epoxide	3956	2344	791
4,4-DDE	14,751	8741	2950
Dieldrin	574	340	115
4,4-DDD	8306	4922	1661
4,4-DDT	12	7	2
Methoxychlor	53	32	11

HQ_0–6:_ Age group 0–6 years, HQ_7-17_: Age 7–17 years, HQ_adt_: For adult.

**Table 6:** Individual average daily dose (ADD), Life average daily dose (LADD), and Cancer risk of OCPs in summer and autumn.

OCPs	ADD_0–6_ × 10^−6^	ADD_7–17_ × 10^−6^	ADD_adt_ × 10^−6^	LADD_0–6&adt_ × 10^−6^	LADD_7–17_ × 10^−6^	Cancer Risk × 10^−13^
α-BHC	269	159	54	231	25	10
γ-BHC	39	23	8	33	4	1.5
β-BHC	618	366	124	530	58	24
Heptachlor	28	16	6	24	3	1.1
δ-BHC	22	13	4	18	2	0.8
Aldrin	60	36	13	52	6	2.3
Hept. Epoxide	51	30	1	44	5	2.0
Endosulfan I	54	32	12	46	5	2.1
4,4-DDE	44	26	9	38	4	1.7
Dieldrin	29	17	6	25	3	1.1
Endrin	0	0	0	0	0	0
4,4-DDD	75	44	15	64	7	2.9
Endosulfan II	36	22	7	31	3	1.4
4,4-DDT	58	34	11	50	5	2.2
Endrin Alde.	748	443	150	641	70	28
Endo. Sulfate	94	55	19	80	87	3.6
Methoxychlor	266	158	53	228	25	10

ADD_0–6_: Age group 0–6 years, ADD_7–17_: Age 7–17 years, ADD_adt_: For adult, LADD_0–6_: Age group 0–6 years, LADD_7–17_: Age group 7–17 years, LADD_adt_: Adult. ADD and LADD are in mg/kg/day.
